# Electrochemical cell for synchrotron nuclear resonance techniques

**DOI:** 10.1107/S1600577524007148

**Published:** 2024-08-16

**Authors:** Sergey Yaroslavtsev, Jean-Philippe Celse

**Affiliations:** a ESRF – The European Synchrotron, CS40220, 38043Grenoble Cedex 9, France; RIKEN SPring-8 Center, Japan

**Keywords:** cell design, electrochemistry, nuclear resonance techniques, Mössbauer spectroscopy, synchrotron radiation

## Abstract

Developing new materials for Li-ion and Na-ion batteries is a high priority in materials science. Such development always includes performance tests and scientific research. Synchrotron radiation techniques provide unique abilities to study batteries. Electrochemical cell design should be optimized for synchrotron studies without losing electrochemical performance. Such design should also be compatible with *operando* measurement, which is the most appropriate approach to study batteries and provides the most reliable results. The more experimental setups a cell can be adjusted for, the easier and faster the experiments are to carry out and the more reliable the results will be. This requires optimization of window materials and sizes, cell topology, pressure distribution on electrodes *etc.* to reach a higher efficiency of measurement without losing stability and reproducibility in electrochemical cycling. Here, we present a cell design optimized for nuclear resonance techniques, tested using nuclear forward scattering, synchrotron Mössbauer source and nuclear inelastic scattering.

## Abstract 2

A new three-electrode battery cell designed for synchrotron techniques, allowing operation in both transmission and reflection geometry, has been tested and demonstrated.

## 1. Introduction

An *ex situ* approach for studying batteries is the most widely used because it is not hindered by any difficulties with the sample environment during measurements. However, this approach has a number of drawbacks: (1) the sample could react with the environment, even oxidize due to air exposure; (2) any meta-stable states that can exist during the charging/discharging processes will disappear before the sample will be measured; (3) the sample may require some treatment (drying, cleaning, *etc*.) after cycling but before measurements, which could influence the end result. The key to improve materials design and to obtain better performance is to understand the charging/discharging processes that *ex situ* studies cannot always access. An *in situ* approach solves some of these problems, but still assumes that every process is under quasi-equilibrium, which is usually only true at low cycling rates. Thus, a comprehensive study requires *operando* measurements, requiring a special cell that could be used during experimental techniques.

Mössbauer spectroscopy has been demonstrated to be an accurate and reliable way to study batteries (Lippens, 2021 ▸; Mikhlin *et al.*, 2021 ▸; Ali *et al.*, 2023 ▸; Yaroslavtsev *et al.*, 2020 ▸). However, studies where measurements were carried out in *operando* mode are limited (Perea *et al.*, 2012*a* ▸,*b* ▸; Brisbois *et al.*, 2016 ▸; Fehse *et al.*, 2019 ▸; Wang *et al.*, 2024 ▸), and were only at low current and/or long-term averaging. One of the main problems of these experiments is cell design. Conventional Mössbauer spectroscopy (CMS) requires the user to locate the detector close to the radioactive source, and the sample should have a relatively large area (≳1 cm in diameter is better). This means that the cell should be flat with a large window, making it difficult to create uniform pressure over the sample which should also be of uniform thickness. Utilizing synchrotron radiation techniques removes certain limitations of the cell design and experimental setup without losing intensity owing to the small beam size. For example, on ID14 at ESRF, the unfocused beam size is ∼0.5 mm × 0.6 mm for energy domain and time domain Mössbauer spectroscopy.

There are many cells made for X-ray diffraction (*e.g. * Nakanishi *et al.*, 2014 ▸) that cannot be used for transmission geometry. In this article, we present a new electrochemical cell designed to be used for nuclear resonance techniques. The pressure over the sample is more homogeneous and the solid body prevents any additional vibrations (which is important for Mössbauer studies). This is advantageous compared with the widely used bag-cell (‘coffee-bag’) design (Villevieille *et al.*, 2014 ▸) or the modified coin-cell design (Tan *et al.*, 2018 ▸). Swagelok-type cells (Tan *et al.*, 2018 ▸; Diaz-Lopez *et al.*, 2020 ▸) are assumed to have a beam parallel to the electrode, which creates a problem – or even makes it impossible – to adjust the sample thickness along the beam, which is crucial especially for synchrotron Mössbauer source (SMS). Compared with one of the most popular cells for synchrotron techniques (Leriche *et al.*, 2010 ▸), the one presented here has a reliable third electrode which allows it to proceed with fast cycling more accurately. The cell was tested with lithium iron phosphate LiFePO_4_ (LFP) based materials against metallic lithium to utilize different nuclear resonance techniques.

Synchrotron nuclear resonance beamlines expand research possibilities by providing several different techniques (Rüffer & Chumakov, 2020 ▸). Three of the most popular are SMS, nuclear forward scattering (NFS) and nuclear inelastic scattering (NIS). SMS allows the user to measure energy domain ^57^Fe Mössbauer spectra (Potapkin *et al.*, 2012 ▸) similar to CMS with a radioactive source. SMS is available at ESRF, SPring-8 and recently at DESY. CMS can provide information about Fe local states, including oxidation states and polyhedra asymmetry, allowing us to distinguish even tiny distortions in the nearest environment. NFS provides similar information (with the exception of central shift absolute values). It has two main advantages: (1) it can measure not only ^57^Fe but a wide range of isotopes (Rüffer & Chumakov, 1996 ▸; Sergueev *et al.*, 2007 ▸); (2) it has almost zero background, allowing faster measurements compared with SMS. Unfortunately, it is more complicated to process spectra from NFS due to the interference between components and correlations between lines splitting and effective thickness (the saturation effect). Thus, this technique requires more rigorous data analysis. However, in the case of the ^57^Fe isotope, to simplify the analysis of the NFS data, one can use information obtained using SMS or CMS. Both SMS and NFS utilize transmission geometry. NIS is a completely different technique that allows extraction of the partial (isotope selective) phonon density of states and material properties associated with it such as the Lamb–Mössbauer factor, force constant, entropy, internal energy and heat capacity. Each peak in the NIS spectrum corresponds to the specific vibration mode of the studied isotope. Thus, for example, if the spectrum contains well distinguished peaks then even without any fitting one can follow its evolution to understand what happens with the rigidity of the corresponding bond. NIS experiments are typically carried out by utilizing the radiation incident on the sample at a grazing angle and measuring the radiation re-emitted from the sample. Examples of nuclear resonance applications to study battery materials can be found in the literature (Giefers *et al.*, 2006 ▸; Wang *et al.*, 2021 ▸; Segi *et al.*, 2016 ▸; Tracy *et al.*, 2014 ▸; Fehse *et al.*, 2019 ▸; Stenina *et al.*, 2024 ▸; Yaroslavtsev & Muller, 2024 ▸).

## 2. Cell design

**Figure 1:**
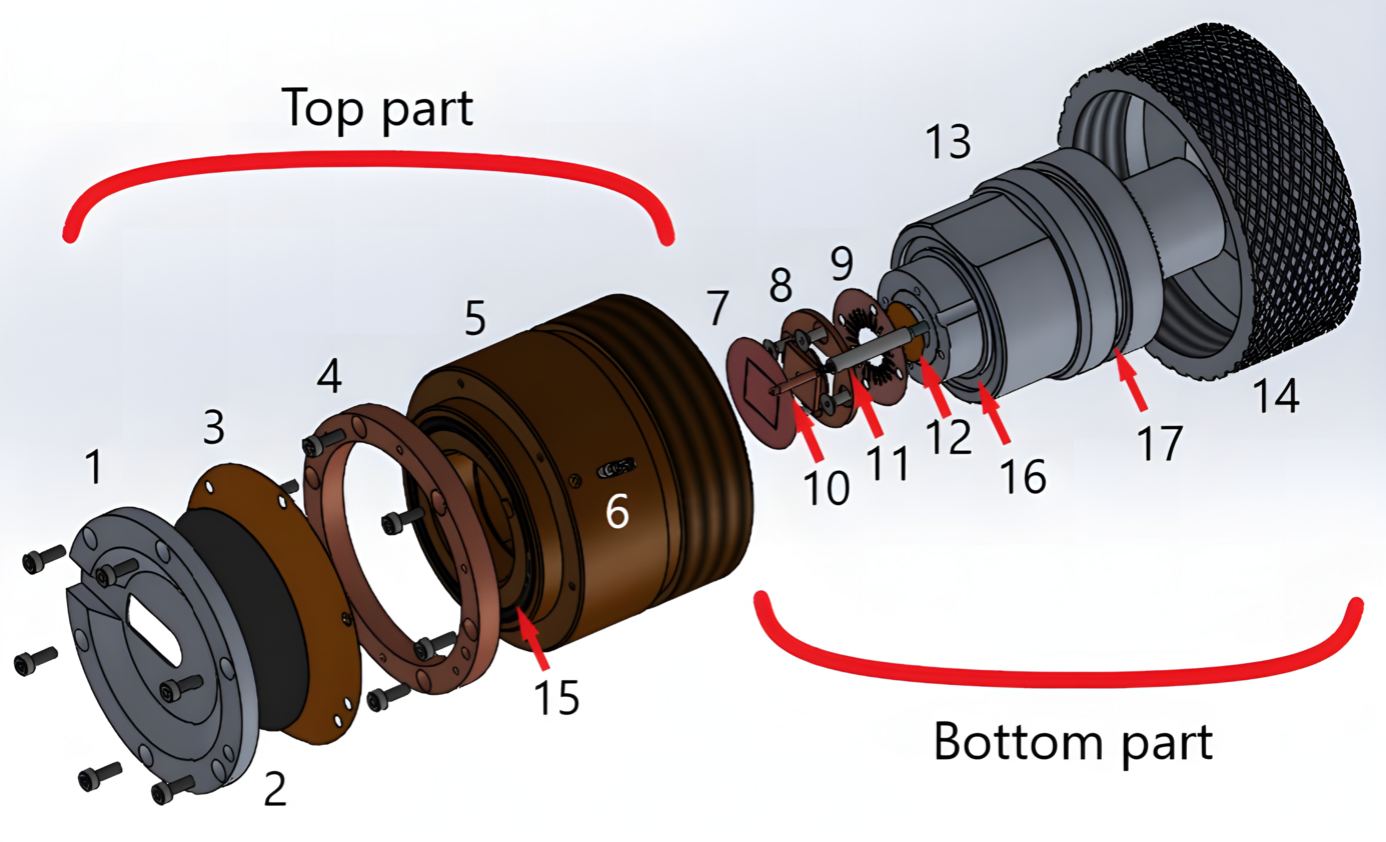
Scheme of the cell. The top part consists of (1) a metal window frame, (2) a carbon glass window, (3) Kapton and aluminium foils, (4) a copper ring, (5) the main body made of PEEK, (6) a screw-connector to the third electrode. (7) The sample battery ‘sandwich’ (cathode/separator/anode). The bottom part consists of (8) a copper/aluminium flat connector, (9) a flat spring, (10) a third-reference electrode connector, (11) a plastic holder with a spring inside for the third electrode, (12) glued Kapton foil, (13) the main body made of steel, (14) a clamping nut with a Teflon circle inside. (15)–(17) Grooves for rubber rings.

The main body consists of two parts that are in contact with the electrodes (see Fig. 1 ▸). The top part is mainly made of PEEK (polyether ether ketone) plastic with a copper ring on top. The window is pressed to the copper ring against a rubber ring by screws (see Fig. 1 ▸) which provide good contact. One of the best options for the window material in such a device is carbon glass which is conductive and rigid to create a homogeneous pressure. If used, electrolytes or electrode materials could react with carbon during cycling; the window could be additionally covered with a Kapton layer along with pure aluminium thin foil to prevent direct contact with the carbon glass. We tried aluminium foil down to 15 µm which still provides good contact (self-resistance of the cell is less than 0.1 Ω and is not too fragile to tear during assembly. The top part also has a side screw hole leading to the third electrode. This hole is also used to release the gas out of the cell while connecting the two parts of the cell together.

The bottom part is made of steel. On top there is a glued Kapton window, brass flat spring and connector plate in two pieces (see Fig. 1 ▸). The connector plate used for Li-ion batteries is made of copper but could be easily changed to an aluminium analog or any other type (without changing every other part) to avoid chemical reactions with the electrode – there are different recommendations depending on what type of anode/cathode is used. Also, the connector plate could have a different height in order to have some kind of hard limit preventing over-pressing against the top window. The flat spring is used for two main reasons: (1) to prevent damage to the top window due to variation in the height of samples and (2) to redistribute the pressure over the sample to be more homogeneous. There is a rubber ring which is pressed between cell parts, but this one could not be pressed well enough because the main pressure should be between the cathode and the anode. Hence there is another rubber ring that squeezes between the walls of the cell parts. In addition, there is also a Teflon circle plate at the bottom which is pressed between two metal surfaces.

The third electrode connector is isolated from the bottom body by a PEEK cover, inside which there is a small spring and metal rod. The third electrode in this case lies against the top electrode, canceling the polarization effect and voltage drop due to the self-resistance of the bottom electrode while measuring the voltage. When the cell is assembled, the outer screw with a Teflon isolation fixes this rod, providing an outer connection for the third electrode. Thus, the cell is sealed and, to minimize assembly time, all cables can be connected to the cell already outside the glovebox.

**Figure 2:**
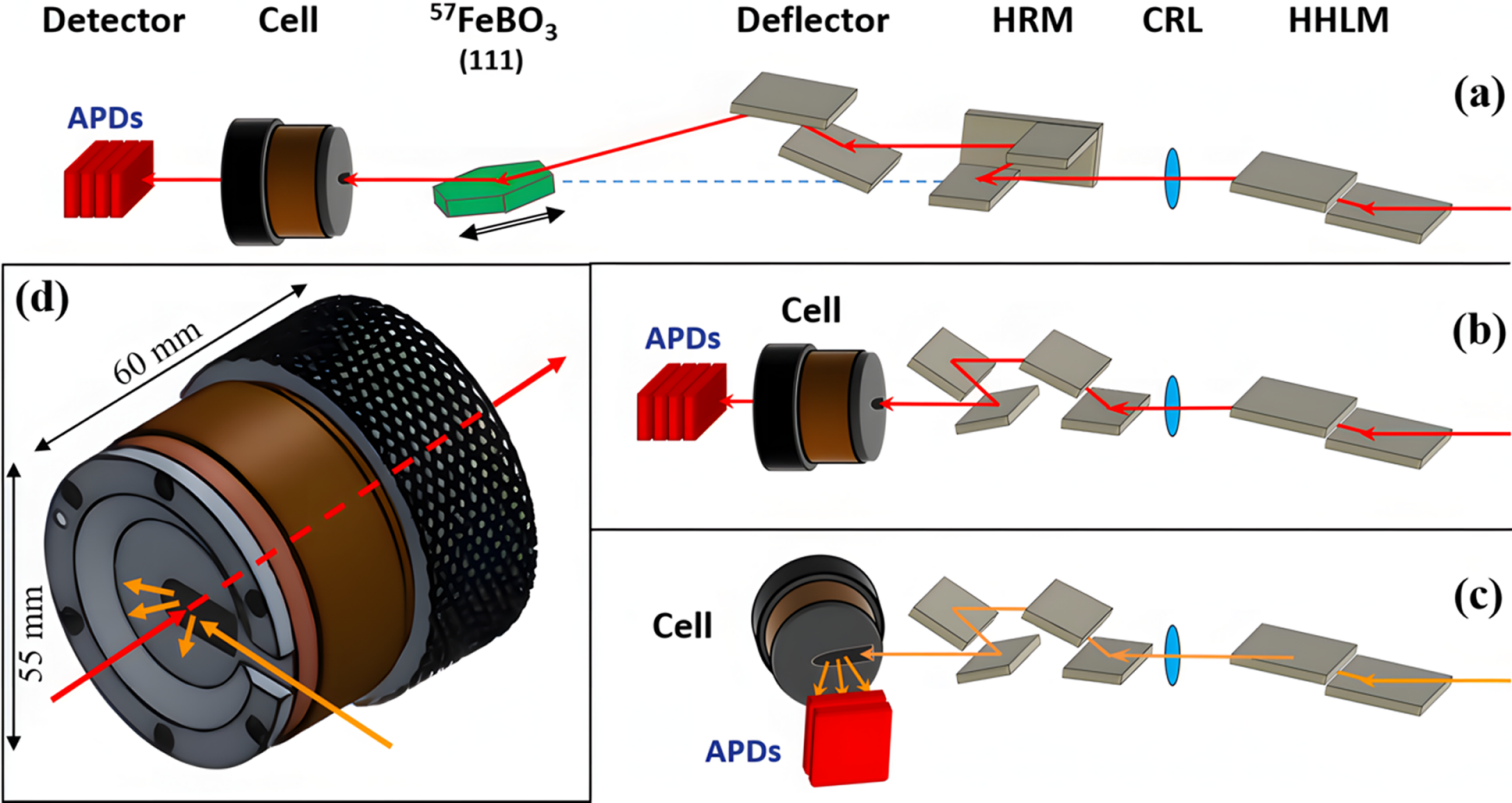
Simplified representation of the optical scheme and cell arrangement in the case of (*a*) SMS, (*b*) NFS, (*c*) NIS. HHLM – high heat load monochromator, HRM – high-resolution monochromator, CRL – compound refractive lens. (*d*) Scheme of the beam path relative to the cell. Red arrows indicate the beam path in transmission geometry (SMS, NFS) and orange arrows indicate the beam path in reflection geometry (NIS).

The bottom outlet window is relatively small (0.8 mm diameter) because it is used only for transmission geometry (see Figs. 1 ▸ and 2 ▸). The top window should be bigger for use with the NIS technique. In this case, the beam is incident on the sample at a grazing angle in order to decrease the absorption of re-emitted radiation by the sample itself, this is why the window should have a prolonged shape (Fig. 2 ▸). Then, the radiation is re-emitted nearly in 2π, which is why the window should be large enough to cover the accepted solid angle of the detector located as close to the window as possible. In the ^57^Fe-NIS case, re-emitted radiation has two energies of about ∼6.4 keV and ∼14.4 keV. For such experiment, the studied sample should be the top electrode, otherwise: (1) the spectrum will contain information about both electrodes, (2) 6.4 keV quanta will be absorbed by the top electrode and (3) the incidence angle will be steeper which means higher self-absorption. The carbon glass window is fragile, which is why the hole in the upper metal part could not be enlarged further; however, in this cell the window opening is 8 mm × 15 mm in the top part (the carbon glass is circular with a diameter of 40 mm) which is large enough for efficient collection of NIS spectra. All tests were carried out using this window; however, the upper metal part could be easily changed to have a smaller opening window down to the beam size for utilizing in transmission geometry only, in which case the carbon glass will not be as necessary.

All springs, windows *etc.* could be assembled in advance outside the glovebox before the experiment. Thus, during the experiment, the user will only need to put inside the electrodes and the separator wet with electrolyte, then attach the top part of the cell to the bottom and finally attach the screw-contact for the third electrode. This assembly could be done in less than 30 min, which is fast compared with the typical cycling time for one sample.

## 3. Electrochemical performance tests

Tests were carried out with a carbon glass window and a bottom connector plate made of copper. For battery assembly, we used EC/EMC/DMC (ethylene carbonate, ethyl methyl carbonate, dimethyl carbonate) in a 1:1:1 ratio and *celgard* (membrane battery separator). The anode material was pure lithium metal. The anode was always at the bottom part of the cell. The top part has extra Kapton and aluminium foils to imitate the worst-case scenario. The cathode material was LiFe_1−*y*_Mn_*y*_PO_4_ (LFP doped with Mn) synthesized by the sol-gel method as outlined in the work of Yaroslavtsev *et al.* (2020 ▸). It was pasted in advance on 15 µm-thick aluminium foil, cut into 10 mm × 10 mm squares. For cycling, a Bio-Logic SP300 potentiostat was used.

**Figure 3:**
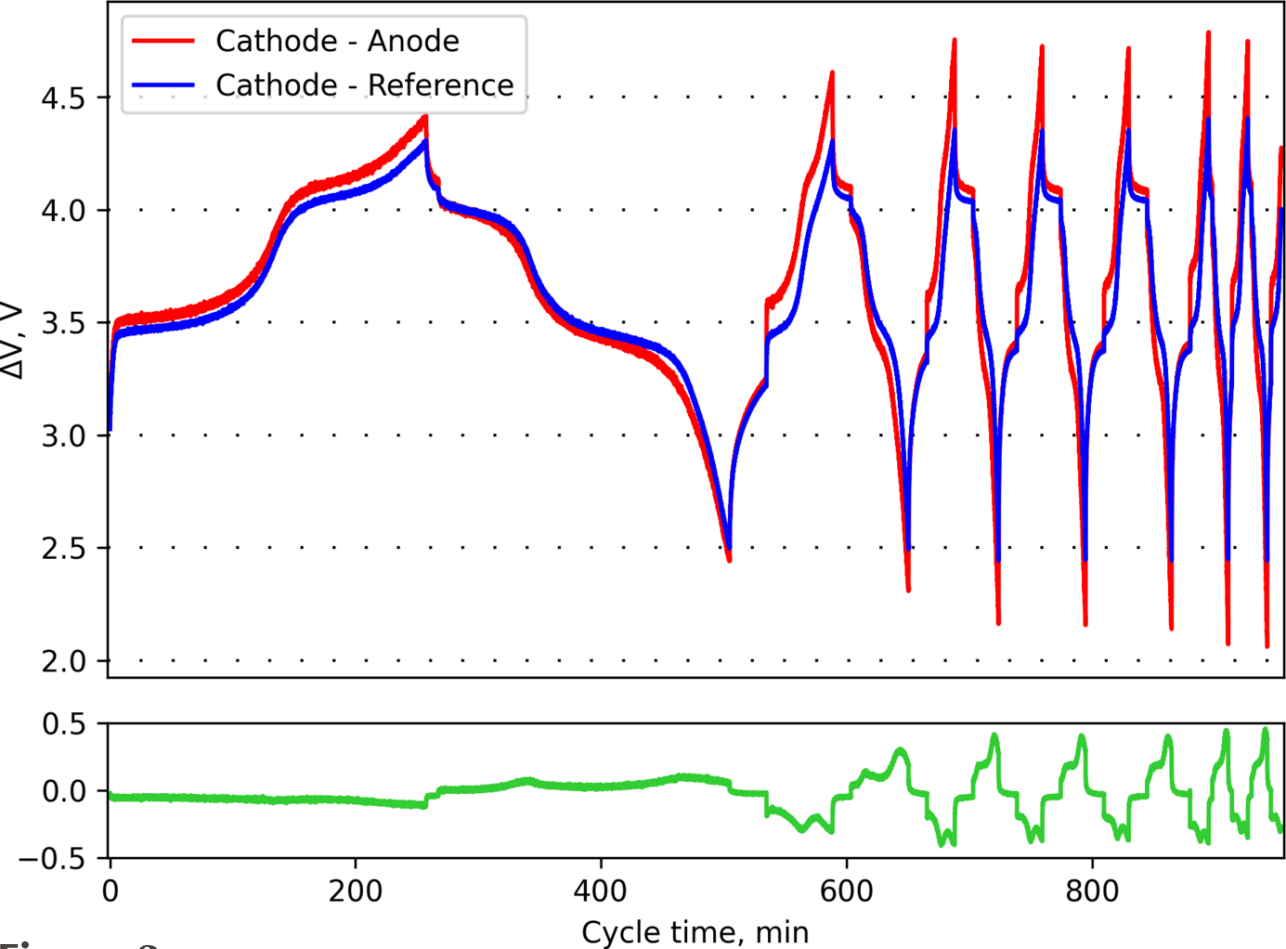
Charge/discharge curves for LiFe_0.5_Mn_0.5_PO_4_. Potential differences for the cathode reference (blue), cathode–anode (red) and their difference (green). Cycles were carried out at rates of ∼C/4, 1C, 2C and 3C.

With the exception of very first cycle^1^ (not shown), all other cycles were reproducible, which supports no continuous irreversible chemical reactions taking place inside the cell. Electrochemical curves of charging and discharging processes at a ∼C/4 cycling rate (1C is the current required to charge or discharge full capacity in 1 h) have nice profiles with two visible plateaus (see Fig. 3 ▸) corresponding to Fe^2+^ ↔ Fe^3+^ and Mn^2+^ ↔ Mn^3+^ transitions. No extra features were observed. Increasing the cycle rate results only in less capacity without losing the representative curve shape. Discharge capacities for cycles with the same rate are equal within error, which indicates not only the good quality of the material but also the good assembly of the cell as a whole (good contacts, uniform pressure, low self-resistance *etc.*). The longest test with one sample was done over three days. Thus, each cell assembly could be used at least for three days (or more) which is enough for a typical synchrotron experiment. In practice, samples will probably be changed every 1 or 2 days.

The differences in voltage measured between two main electrodes and between the cathode and the third electrode (reference) are shown in Fig. 3 ▸. The differences are more pronounced at high cycling rates. This clearly demonstrates the benefit of the third electrode to be able to test batteries up to maximum performance especially at high currents. The three-electrode scheme is optimal for most experiments; implementation of a fourth electrode would make the preparation process and the cell design overcomplicated and bring no significant improvements.

## 4. Measurement tests

All test measurements were performed at the nuclear resonance beamline (Rüffer & Chumakov, 1996 ▸) ID18 (recently moved to ID14) at ESRF. We utilized nuclear resonance techniques to study Fe local states in the material and therefore there should be no Fe-containing elements in the beam path^2^. For this cell, the beam passes through carbon glass, Kapton foil and aluminium foil. It is easy to buy all of these materials without Fe impurity. To achieve better performance, the electronic absorption should be as low as possible. Owing to low thickness, the windows used absorb altogether about 5% of 14.4 keV radiation.

**Figure 4:**
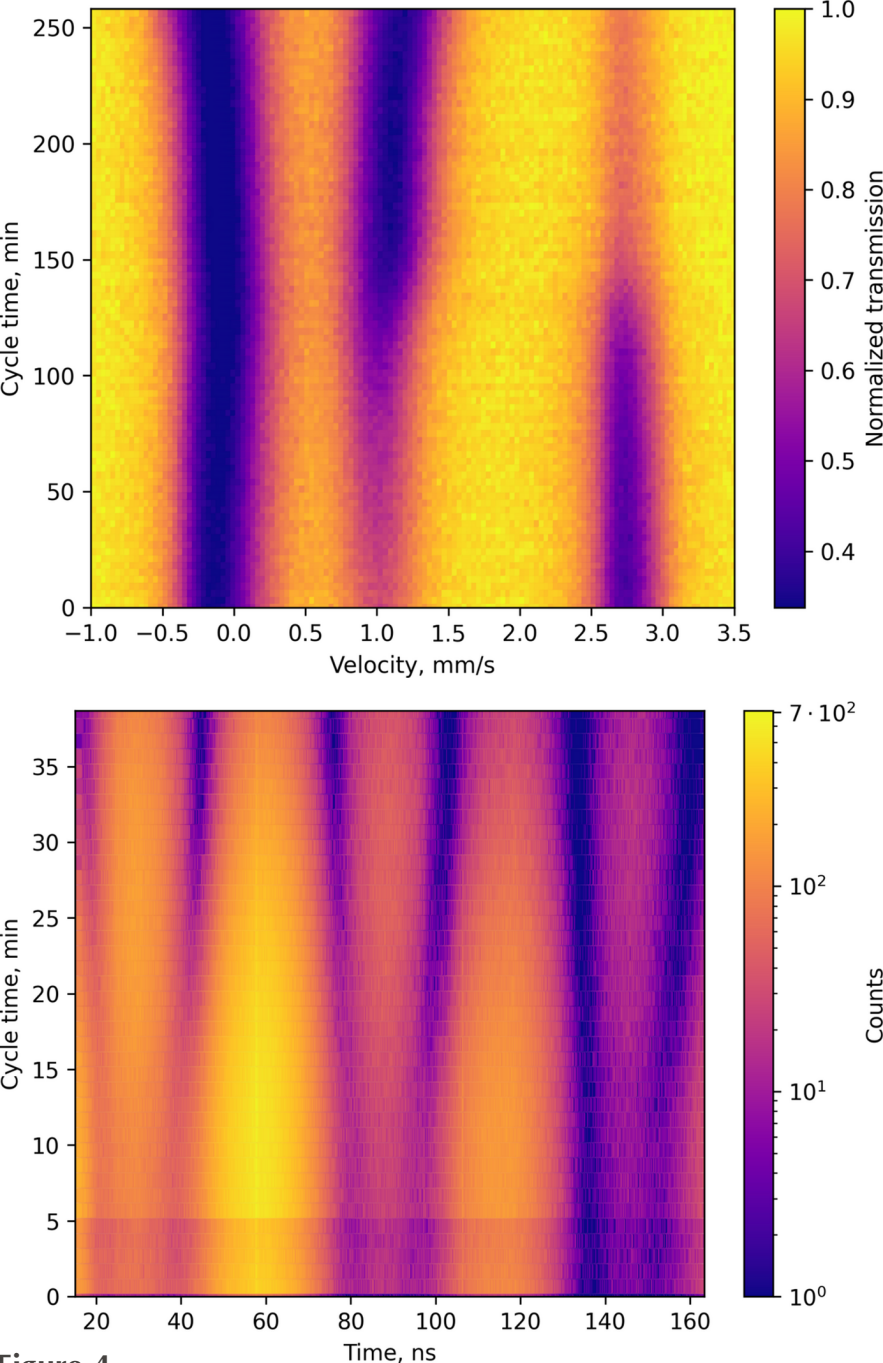
Evolution of SMS spectra during the charging process (top); evolution of the NFS spectra during the discharging process (bottom).

For partially enriched (15% of ^57^Fe) samples with optimized thickness, we managed to collect reasonable spectra within 3 min for SMS and within 1 min for NFS (see Figs. 4 ▸, 5 ▸ and 6 ▸). The measurement time for one spectrum depends on the spectrum complexity and the features of interest in a particular study. The optimized sample, together with a new cell, allow us to study the dynamics of the cycling process by following the evolution of the spectra (Fig. 4 ▸). Collecting a spectrum every few minutes allows us to follow even small changes. Even without any fitting, it is clear how resonance lines shift during the charging process, meaning continuous change of hyperfine parameters.

**Figure 5:**
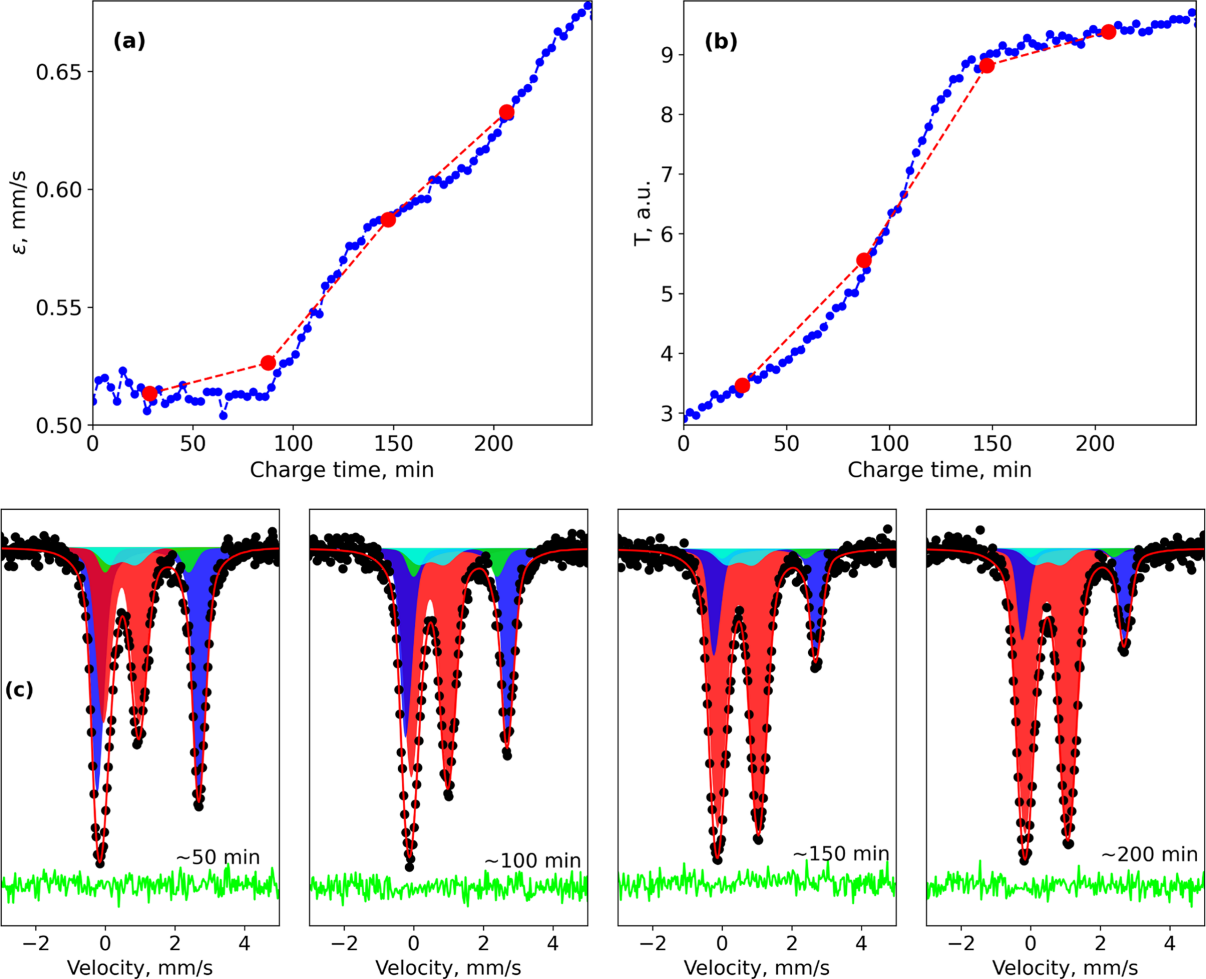
Evolution of the (*a*) quadrupole shift and (*b*) effective thickness of the Fe^3+^ component in Li_*x*_Fe_0.5_Mn_0.5_PO_4_ during the charging process. Red points indicate averaging within 1 h. (*c*) Representative spectra (measured for 3 min each) with fitting at different stages of the charging process.

Let us consider the charging process for Li_*x*_Fe_0.5_Mn_0.5_PO_4_^3^ (Fig. 4 ▸, left). The fitting of spectra measured with SMS was done using the *SYNCmoss* software (Yaroslavtsev, 2023 ▸), which was developed to take into account the specific shape of the SMS instrumental function. Effective thicknesses (proportional to Fe fractions) of components could be found as a result of fitting because *SYNCmoss* can calculate the full transmission integral. There is a clear step in the evolution of Fe^3+^ effective thickness [Fig. 5 ▸(*b*)]. It indicates a switch from an iron oxidation process (Fe^2+^ → Fe^3+^) to mainly a manganese oxidation process (Mn^2+^ → Mn^3+^). Moreover, one can see that even the first half of the observed dependency is not linear, which means that manganese partially participates even at the beginning of the charge. Even more interesting is the behavior of the quadrupole shift (ɛ) of the Fe^3+^ component [Fig. 5 ▸(*a*)]. There are several steps indicating that there are priorities, whereby Fe ions are oxidized first. In previous work, Yaroslavtsev *et al.* (2020 ▸) proposed that these priorities depend on the second-nearest environment of the Fe ions. However, due to the new data, it is obvious that the cycling process is even more complicated. Details of the fitting model and discussion of the cycling process mechanism for LiFe_1−*y*_Mn_*y*_PO_4_ were recently published (Stenina *et al.*, 2024 ▸).

Measuring each individual spectrum for more than 1 h as is typically done in CMS (Brisbois *et al.*, 2016 ▸; Mahmoud *et al.*, 2018 ▸; Perea *et al.*, 2012*a* ▸,*b* ▸; Wang *et al.*, 2024 ▸) would hide all features mentioned above (Fig. 5 ▸). Another problem of averaging (within 1 h or longer) could be the shape of spectra. In the case presented, hyperfine parameters change during cycling, leading to changes not only in resonance line intensities but also in their positions. Thus, averaging would lead to each resonance line shape being asymmetric and unable to be fitted with an individual single line, creating a problem with data processing.

**Figure 6:**
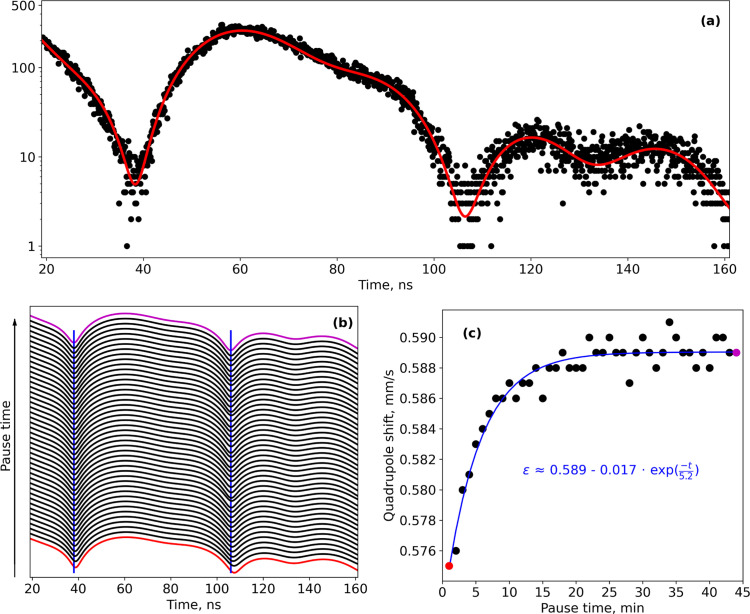
(*a*) Typical NFS spectrum with a fitting curve (red). (*b*) Evolution of the NFS spectrum fitting curve during a pause in electrochemical cycling. Blue vertical lines are guidelines for positions of local minima. (*c*) Evolution of quadrupole shift of the Fe^3+^ component. The blue line is an exponential fit. Red and purple indicate the beginning and the end of the pause.

The NFS technique could provide similar information to SMS, but measurement time could be reduced even more. However, as mentioned before, without knowing the exact model, the fitting procedure could be problematic. Thus, to obtain a more unambiguous result, one can combine NFS with SMS or CMS. Knowing the model for Li_*x*_Fe_0.5_Mn_0.5_PO_4_ from SMS measurements, we applied it to NFS spectra. Fitting of the NFS spectra was also done using the *SYNCmoss* software (Yaroslavtsev, 2023 ▸) in ‘logarithmic’ mode. Measurements during pauses in cycling (Fig. 6 ▸, pauses were done in an open circuit voltage regime) allow us to observe the relaxation processes that occur inside the material. The uncertainty of each quadrupole shift value obtained from the fitting is about ∼0.002 mm s^−1^. Of course absolute values have bigger errors (due to uncertainties in calibration, model, *etc*.), but they do not affect uncertainties in differences between values obtained. The maximum difference of quadrupole shift during relaxation is more than ∼0.01 mm s^−1^. Thus, the lifetime could be estimated from the evolution of the quadrupole shift. The shortest lifetime observed is estimated to be ∼5 min [Fig. 6 ▸(*c*)]. Details of this experiment and an explanation of the relaxation phenomena were recently published [Yaroslavtsev & Muller, 2024 ▸].

**Figure 7:**
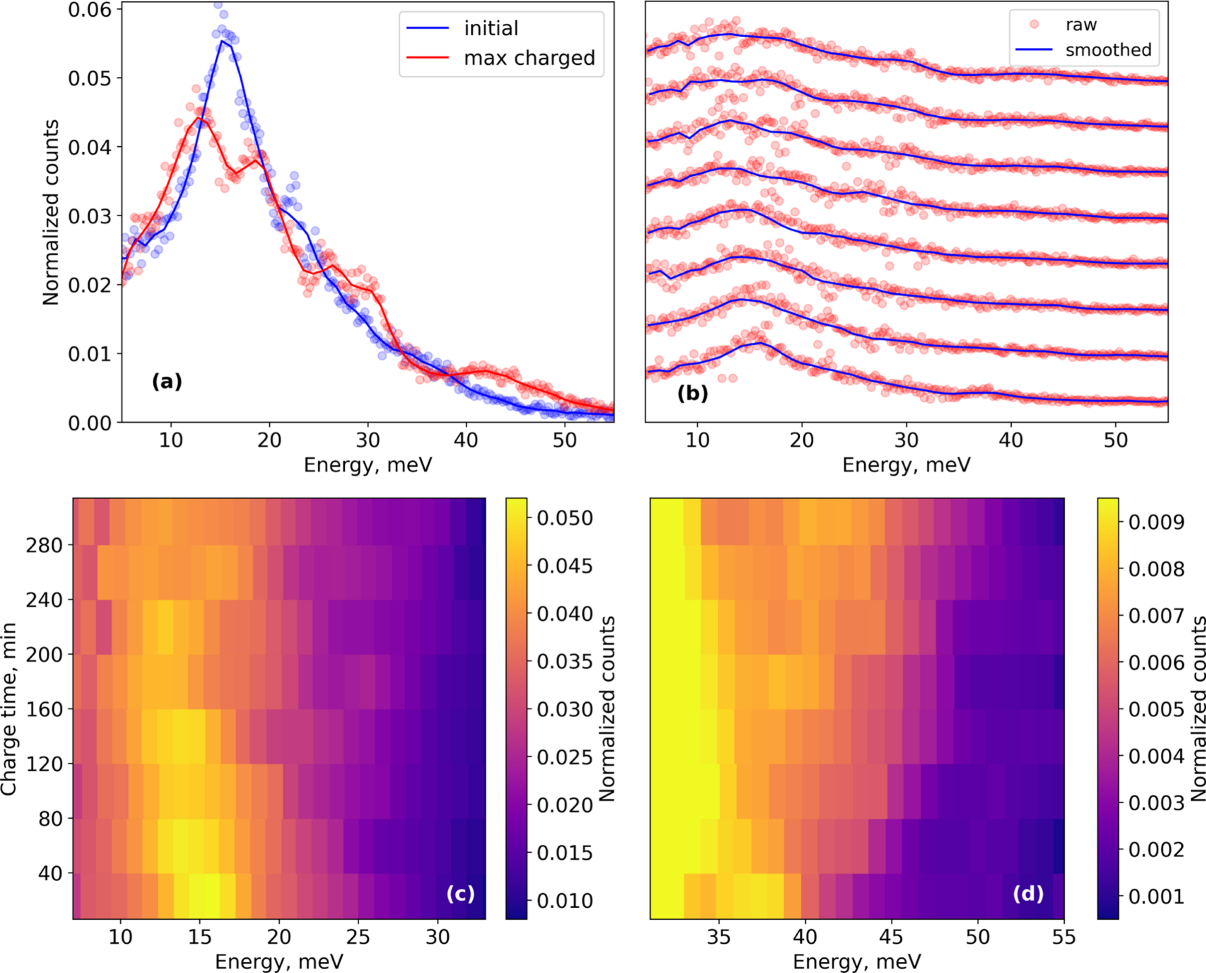
(*a*) Energy dependencies of NIS for initial (blue) and maximally charged (red) Li_*x*_Fe_0.8_Mn_0.2_PO_4_ samples measured for ∼3 h. (*b*) Energy dependencies measured in *operando* mode, ∼35 min per measurement. Points – raw data; solid lines – smoothed data. (*c*, *d*) Color map representations of smoothed data for the two energy ranges.

Adapting the cell to the NIS technique poses the greatest challenge because of high absorption of 6.4 keV quanta and because radiation from the sample is emitted in 2π. In the setup used, about 50% of 6.4 keV quanta [it is not just windows that absorb radiation, but at least half of the absorption is related to the sample itself and the electrode aluminium foil (15 µm)] and more than 90% of 14.4 keV quanta re-radiated from the sample towards the detector reach it. For the LFP based sample with 15% of ^57^Fe enrichment, to extract quantitative information from the NIS energy dependency, collection should take ∼3 h. However, for the qualitative demonstration test, we tried to proceed with an *operando* experiment with 35 min per measurement.^4^ Binning and smoothing were applied to the data collected (Fig. 7 ▸) which allowed us to distinguish two trends. During the charging process, the main peak around 15 meV became broader and a new peak at ∼44 meV appeared [Figs. 7 ▸(*c*) and 7 ▸(*d*)]. These trends match the differences between the energy dependencies of NIS for initial and maximally charged samples measured for a longer time [Fig. 7 ▸(*a*)]. Of course due to poor statistics these results could not be used to evaluate the data processing required to extract quantitative results. To improve the signal-to-noise ratio within *operando* experiments, one can prepare samples with 100% ^57^Fe enrichment. Moreover, test measurements were carried out at ESRF during EBS commissioning time in 16-bunch mode (timing mode) used for NFS and NIS techniques with only 30 mA current in the storage ring, which will be increased up to 90 mA in future (at the time of writing, it is about 70 mA) which also decreases the measurement time by a corresponding factor (the count rate is proportional to current in a storage ring). Thus, for an optimized experiment, reasonable data can be collected in 30 min, which is already acceptable for *operando* studies with currents of ≲C/5.

## 5. Conclusions

A new electrochemical cell for nuclear resonance techniques has been designed. Using this cell, SMS, NFS and NIS spectra can be collected efficiently with a high count rate (low absorption by cell windows). At the same time, this new cell provides stable cycling even at high currents, and it does not lose electrochemical performance even after several days of usage. The step shape of the charge/discharge curves indicates homogeneous cycling over the sample even at high current, which can be attributed to uniform pressure over the sample. It was shown that well optimized sample studies can be carried out in *operando* mode for SMS, NFS and NIS. Fast *operando* Mössbauer studies of batteries will bring a new perspective as well as additional information about the charging/discharging processes. The new cell could also be used for other non-nuclear resonance techniques in both transmission and reflection geometries.

## Footnotes

1 The specificity of the first cycle is related to the formation of the solid electrolyte interphase and the sample itself, which is typical for electrochemical cycling of Li versus LiFePO_4_.

2 NFS and NIS also could also be used with other isotopes. The windows do not contain any of the isotopes for which the spectra could be measured with these approaches.

3 Material characterization techniques (X-ray diffraction, scanning electron microscopy) reveal only one orthorhombic phase with no impurities and no texture. Details can be found in the work of Stenina *et al.* (2024 ▸).

4 Beamline optics adjustments were required between measurements, leading to gaps of several minutes in between. To decrease charge inhomogeneity within the measurement time, each was done as two sequences in a row.

## References

[bb1] Ali, G., Akbar, M., Iftikhar, F. J., Wali, Q., Kalska Szostko, B., Satuła, D. & Chung, K. Y. (2023). *J. Energy Chem.*, **77**, 535–542.

[bb2] Brisbois, M., Caes, S., Sougrati, M., Vertruyen, B., Schrijnemakers, A., Cloots, R., Eshraghi, N., Hermann, R., Mahmoud, A. & Boschini, F. (2016). *Solar Energy Mater. Solar Cells*, **148**, 67–72.

[bb4] Diaz-Lopez, M., Cutts, G. L., Allan, P. K., Keeble, D. S., Ross, A., Pralong, V., Spiekermann, G. & Chater, P. A. (2020). *J. Synchrotron Rad.* **27**, 1190–1199. 10.1107/S160057752000747X PMC7467346 32876593

[bb5] Fehse, M., Bessas, D., Darwiche, A., Mahmoud, A., Rahamim, G., La Fontain, C., Hermann, R., Zitoun, D., Monconduit, L., Stievano, L. & Sougrati, M. (2019). *Batteries Supercaps*, **2**, 66–73.

[bb6] Giefers, H., Koval, S., Wortmann, G., Sturhahn, W., Alp, E. & Hu, M. (2006). *Phys. Rev. B*, **74**, 094303.

[bb7] Leriche, J., Hamelet, S., Shu, J., Morcrette, M., Masquelier, C., Ouvrard, G., Zerrouki, M., Soudan, P., Belin, S., Elkaïm, E. & Baudelet, F. (2010). *J. Electrochem. Soc.* **157**, A606–A610.

[bb8] Lippens, P.-E. (2021). *Modern Mössbauer Spectroscopy: New Challenges Based on Cutting-Edge Techniques*, ch. 7, pp. 319–379. Springer.

[bb9] Mahmoud, A., Karegeya, C., Sougrati, M. T., Bodart, J., Vertruyen, B., Cloots, R., Lippens, P. & Boschini, F. (2018). *Appl. Mater. Interfaces*, **10**, 34202–34211. 10.1021/acsami.8b10663 30216721

[bb10] Mikhlin, Y., Likhatski, M., Bayukov, O., Knyazev, Y., Velikanov, D., Tomashevich, Y., Romanchenko, A., Vorobyev, S., Volochaev, M., Zharkov, S. & Meira, D. (2021). *ACS Omega*, **6**, 7533–7543. 10.1021/acsomega.0c06052 PMC7992167 33778265

[bb11] Nakanishi, K., Kato, D., Arai, H., Tanida, H., Mori, T., Orikasa, Y., Uchimoto, Y., Ohta, T. & Ogumi, Z. (2014). *Rev. Sci. Instrum.* **85**, 084103. 10.1063/1.4891036 PMC4137878 25173283

[bb12] Perea, A., Sougrati, M., Ionica-Bousquet, C., Fraisse, B., Tessier, C., Aldon, L. & Jumas, J. (2012*a*). *RSC Adv.* **2**, 2080–2086.

[bb13] Perea, A., Sougrati, M., Ionica-Bousquet, C., Fraisse, B., Tessier, C., Aldon, L. & Jumas, J. (2012*b*). *RSC Adv.* **2**, 9517–9524.

[bb14] Potapkin, V., Chumakov, A. I., Smirnov, G. V., Celse, J.-P., Rüffer, R., McCammon, C. & Dubrovinsky, L. (2012). *J. Synchrotron Rad.* **19**, 559–569. 10.1107/S0909049512015579 22713890

[bb16] Rüffer, R. & Chumakov, A. (1996). *Hyperfine Interact.* **97–98**, 589–604.

[bb17] Rüffer, R. & Chumakov, A. (2020). *Synchrotron Light Sources and Free-Electron Lasers*, pp. 2251–2287. Springer.

[bb18] Segi, T., Masuda, R., Kobayashi, Y., Tsubota, T., Yoda, Y. & Seto, M. (2016). *Hyperfine Interact.* **237**, 78.

[bb19] Sergueev, I., Chumakov, A. I., Beaume-Dang, T. D., Rüffer, R., Strohm, C. & van Bürck, U. (2007). *Phys. Rev. Lett.* **99**, 097601. 10.1103/PhysRevLett.99.097601 17931037

[bb20] Stenina, I., Kulova, T. & Yaroslavtsev, A. (2024). *Mater. Today Chem.* **39**, 102160.

[bb21] Tan, C., Daemi, S., Taiwo, O., Heenan, T. M. M., Brett, D. & Shearing, P. (2018). *Materials*, **11**, 2157. 10.3390/ma11112157 PMC6266800 30388856

[bb22] Tracy, S., Mauger, L., Tan, H., Muñoz, J. A., Xiao, Y. & Fultz, B. (2014). *Phys. Rev. B*, **90**, 094303.

[bb23] Villevieille, C., Sasaki, T. & Novák, P. (2014). *RSC Adv.* **4**, 6782–6789.

[bb24] Wang, H., Braun, A., Cramer, S. P., Gee, L. B. & Yoda, Y. (2021). *Crystals*, **11**, 909. 10.3390/cryst11080909 PMC9109880 35582460

[bb25] Wang, Z., Nie, K., Sougrati, M. T., Wang, C., Liu, Z., Wang, J., Ge, R., Zheng, Q. & Wang, J. (2024). *Chem. Eng. J.* **488**, 151090.

[bb27] Yaroslavtsev, S. (2023). *J. Synchrotron Rad.* **30**, 596–604. 10.1107/S1600577523001686 PMC10161888 37000184

[bb28] Yaroslavtsev, S. & Muller, S. (2024). *Mater. Today Chem.* **39**, 102159.

[bb26] Yaroslavtsev, S., Vostrov, N., Novikova, S., Kulova, T., Yaroslavtsev, A. & Rusakov, V. (2020). *J. Phys. Chem. C*, **124**, 13026–13035.

